# Employment of Marine Polysaccharides to Manufacture Functional Biocomposites for Aquaculture Feeding Applications

**DOI:** 10.3390/md13052680

**Published:** 2015-04-29

**Authors:** Marina Paolucci, Gabriella Fasulo, Maria Grazia Volpe

**Affiliations:** 1 Istituto di Scienze dell’Alimentazione—CNR Via Roma 52, 83100 Avellino, Italy; E-Mails: paolucci@unisannio.it (M.P.); gabriella.fasulo@isa.cnr.it (G.F.); 2 Department of Science and Technologies, University of Sannio, Via Port’Arsa, 11, 82100 Benevento, Italy

**Keywords:** functional biocomposites, quercetin release, aquaculture applications, welfare fish

## Abstract

In this study, polysaccharides of marine origin (agar, alginate and κ-carrageenan) were used to embed nutrients to fabricate biocomposites to be employed in animal feeding. The consistency of biocomposites in water has been evaluated up to 14 days, by several methods: swelling, nutrient release and granulometric analysis. Biocomposites were produced with varying percentages of nutrients (5%–25%) and polysaccharides (1%–2%–3%). All possible biopolymer combinations were tested in order to select those with the best network strength. The best performing biocomposites were those manufactured with agar 2% and nutrients 10%, showing the lowest percentage of water absorption and nutrient release. Biocomposites made of agar 2% and nutrients 10% were the most stable in water and were therefore used to analyze their behavior in water with respect to the release of quercetin, a phenolic compound with demonstrated high antibacterial and antioxidant activities. The leaching of such molecules in water was therefore employed as a further indicator of biocomposite water stability. Altogether, our results confirm the suitability of agar as a binder for biocomposites and provide a positive contribution to aquaculture.

## 1. Introduction

Marine organisms synthesize a considerable variety of biopolymers, which can begrouped into three main classes: polysaccharides, proteins and nucleic acids. The exploitation of marine biopolymers for industrial and medical purposes is a fast-growing sector of enormous interest, not only in research, but also in the progress of society, as demonstrated by the increasing number of different types of compounds isolated fromaquatic organisms and transformed into profitable products for health applications and food/feed industry [1,2,3]. Differently from feed for livestock, feed for aquaculture requires an adequate level of processing to guarantee good stability in water, long enough for animals to consume it. Indeed, some species are grazers and need time to eat the feed offered. Thus, in order to facilitate rearing management, the addition of binders to the feed has been considered [4]. Binders are essential for the manufacturing of formulated feed, and research is always on the look-out for new solutions based on eco-friendly, sustainable and cost-effective materials. Carbohydrates are natural biopolymers whose molecular diversity includes structures and characteristics with a large array of functions of great significance, making them suitable candidates as binders. Carbohydrates create three-dimensional networks or hydrogels that entrap nutrients and are sustainable and biodegradable [5]. Carbohydrate binders, mainly polysaccharides, in animal feed have been tested for water stability and growth performance in aquatic species with conflicting results [4]. Consistent results have been obtained regarding improved growth rates in crayfish fed on manufactured pellets [6,7,8].

Agar is an unbranched polysaccharide obtained from the cell walls of some species of red algae, primarily the genera belonging *Gelidium* and *Gracilaria*. Its structure consists of a gelling fraction, agarose and other non-gelling portions, agaropectins [9]. To allow the solubilization of agar, the water solution is heated up to 80–85 °C; at this temperature, agar macromolecules are statistically distributed in predominant random coil arrangements. As the sol cools down close to gelling temperature (32–43 °C), macromolecular chains start to organize in a left-handed dual helix structure due to hydrogen bond formation; the macro-grid thus obtained is stable up to 85 °C, the temperature at which it become a sol. Sodium alginate is the sodium salt of alginic acid, the structural component of the intercellular walls of *Phaeophyceae* brown seaweeds. In water solution and in the presence of divalent cations, such as calcium ion, sodium alginate yields water-insoluble gels. The divalent ions strongly interact with the-COO**^−^** groups of the base residual of guluronic blocks forming ionic bridges between different chains [10]. Carrageenan is the hydrocolloid obtained from some red seaweeds [11,12]. Its structure consists principally of sodium, potassium, calcium, magnesium and ammonium sulfate esters of galactose and 3,6-anhydrogalactose copolymers. These hexoses are alternately linked α-1,3 and β-1,4 in the polymer. The content of sulfate groups varies 18%–40%, and according to the number and position of these groups in the units of galactose, it is possible to distinguish different types of carrageenans: kappa-carrageenan (κ-carrageenan), iota-carrageenan (ι-carrageenan) and lambda-carrageenan (λ-carrageenan). κ-Carrageenan contains residues of β-d-galactose-4-sulfate with 1,3 bonds with residues of 3,6-anhydro-α-d-galactose with 1,4 bonds. ι-Carrageenan has a similar structure to κ-carrageenan, with a difference in the degree of sulfation on Carbon 2. λ-Carrageenan contains residues of β-d-galactose-2-sulfate and 1,3 bonds with residues of β-d-galactose-2,6-disulfate bonds with 1,4. κ-Carrageenan is the most suitable to form resistant gels, because the mechanism of gelation is based on the formation of a double helix structure [13]. Quercetins are phenolic compounds (flavonoids) with several hydroxyl groups on aromatic rings [14] with demonstrated high antioxidant activities and also potential antiviral [15], antibacterial [16,17,18] and antiparasitic effects [19,20,21].

In this study, a number of polysaccharide binders of marine origin were mixed with various percentages of nutrients to manufacture biocomposites with prolonged firmness that were then tested for water retention and nutrient release up to two weeks’ immersion in water. Moreover, quercetin, an active compound with antimicrobial and antifungal action, was employed to generate functional biocomposites. In particular, we focused on the application of sodium alginate, κ-carrageenan and agar as binders to manufacture feed for aquatic species.

## 2. Results and Discussion 

### 2.1. Biocomposites Manufacturing and Selection

In this paper, functional biocomposites manufactured with different percentages of marine polysaccharides as binders, nutrients and quercetin were analyzed to determine the binder’s capability to improve the biocomposites’ stability in water.

**Figure 1:**
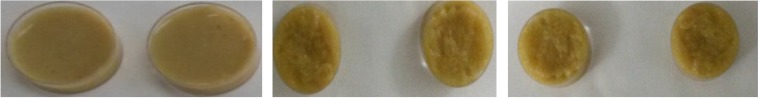
Pictures of biocomposites made with 2% polymer and 10% nutrients. (Right) agar; (center) alginate; (left) κ-carrageenan.

To investigate the water stability, biocomposites manufactured with percentages ranging from 1% to 3% of each one of the following polysaccharides, agar, κ-carrageenan and sodium alginate made with percentages of nutrients ranging from 5% to 25%,were compared according to the network strength graduation used by Pearce *et al.* (2002) [22]. Figure 1 reports pictures of samples of biocomposites cast in small Petri capsules.

Agar-based biocomposites at any percentage had a smooth and glossy surface, while both sodium alginate- and κ-carrageenan-based biocomposites appeared bumpy and dull. In Table 1, the complete list of manufactured biocomposites is reported, along with their consistency. Biocomposites made with agar 2% and 3% and nutrients 5%–10% gave the best results in terms of consistency, followed by agar 1% with all percentages of nutrients tested and agar 2% and 3% with nutrients 15%, 20%–25%. Sodium alginate and κ-carrageenan performed less well at all percentages of polysaccharide and nutrients tested. This is probably due to the different supermolecular structure of the gels. Alginate forms the so-called “egg-box” structure, where the contact points of the polymeric chainsare formed by ionic bonds by means of divalent ions (Ca^2+^). In gels made with κ-carrageenan, the molecules assume a helical shape due to the effects of torsional movements. The association of many chains leads to the formation of multiple helices, whose anionic nature requires the presence of cations, such as Ca^2+^. Thus, with both alginate and κ-carrageenan, the gels formed are rigid, even at low concentrations. Thus, although alginate and κ-carrageenan are well known by their good gel-forming capability, lowconsistent systems of alginate and κ-carrageenan are probably due to interference of the molecules of nutrients with the crosslinking process thatoccurs with calcium ions.The mechanism of agar gel formation is instead temperature dependentand reversible. At the beginning, single and double helices are formed, then individual helices aggregate into multiple helices [23]. Although speculative, we may hypothesize that the slower gelation process occurring in agar gel allowed the nutrients to embed in the network, thus leading to biocomposites with good and very good consistency with respect to gels made of sodium alginate and κ-carrageenan.

Only biocomposites with very good and good consistency were further tested for water uptake and nutrients release being. Thus, agar-based biocomposites made with percentages of nutrients ranging from 5% to 25% were manufactured and further analyzed for water behavior. The proximate composition is shown in Table 2.

**Table 1:** Consistency evaluation according to the terminology used by Pearce *et al.* [22]: 1, very good consistency; 2, good consistency; 3, weak consistency; 4, inconsistent.

No.	Biocomposites	Biopolymer %	Nutrients %	Consistency
1	Sodium Alginate	1	15–20–25	4
2	Sodium Alginate	1	5–10	3
3	Sodium Alginate	2	15–20–25	4
4	Sodium Alginate	2	5–10	3
5	Sodium Alginate	3	15–20–25	4
6	Sodium Alginate	3	5–10	3
7	κ-Carrageenan	1	15–20–25	4
8	κ-Carrageenan	1	5–10	3
9	κ-Carrageenan	2	15–20–25	4
10	κ-Carrageenan	2	5–10	**3**
11	κ-Carrageenan	3	15–20–25	4
12	κ-Carrageenan	3	5–10	3
13	Agar	1	15–20–25	2
14	Agar	1	5–10	2
15	Agar	2	15–20–25	2
16	Agar	2	5–10	1
17	Agar	3	15–20–25	2
18	Agar	3	5–10	1

### 2.2. Biocomposites Water Stability

Water stability of feed is of primary importance in the processing of aquaculture diets; it is greatly influenced by the properties of the binders, although the ingredients themselves have an influence on the characteristics of the binders [24]. Despite the water stability representing a major concern of the aquaculture industry, there is no standard method to determine feed water stability. It is usually estimated by the method of dry matter weight loss, according to which a certain amount of feed, usually in the form of pellets, is placed in a water-containing beaker and allowed to stay for a variable length of time [4]. In the present study, the stability in water of biocomposites was evaluated through the degree of water absorption (swelling test), dry matter loss (nutrients release) and granulometric analysis.

**Table 2:** Proximate analysis of agar (1%–2%–3%)-based biocomposites and nutrients ranging from 5% to 25%.

Agar (%)	Nutrients (%)	Total Lipids (%)	Protein (%)	Carbohydrates (%)	Water (%)
1	5	0.240 ± 1.04	2.56 ± 0.44	7.9 ± 1.12	89.3 ± 0.98
1	10	0.420 ± 1.08	3.06 ± 0.60	14.7± 0.98	81.2 ± 1.01
1	15	0.740 ± 0.98	4.47 ± 0.38	21.7 ± 1.02	73.09± 1.15
1	20	0.975 ± 0.79	5.96 ± 1.02	30.5 ± 0.76	62.6 ± 1.30
1	25	1.58 ± 0.08	7.39 ± 0.79	38.4 ± 0.54	52.6 ± 0.94
2	5	0.257 ± 0.16	2.32 ± 1.12	7.7 ± 0.38	88.3 ± 0.84
2	10	0.432 ± 0.21	3.46 ± 0.74	13.7 ± 0.44	82.2 ± 0.79
2	15	0.750 ± 0.38	4.50 ± 0.67	21.0 ± 0.67	72.8 ± 0.78
2	20	0.946 ± 0.20	5.89 ± 0.21	30.3 ± 1.23	62.1 ± 1.05
2	25	1.64 ± 0.24	7.25 ± 1.02	38.0 ± 1.21	51.9 ± 1.16
3	5	0.235 ± 0.19	2.32 ± 0.14	7.5 ± 1.34	89.1 ± 0.60
3	10	0.453 ± 0.32	3.38 ± 0.23	14.3 ± 0.98	81.7 ± 0.59
3	15	0.736 ± 0.48	4.86 ± 0.36	21.9 ± 0.87	73.32 ± 0.54
3	20	0.967 ± 0.21	6.02 ± 0.16	31.1 ± 1.37	62.3 ± 0.54
3	25	1.59 ± 0.38	7.63 ± 0.76	38.2 ± 0.97	52.1 ± 0.38

#### 2.2.1. Swelling

**Figure 2:**
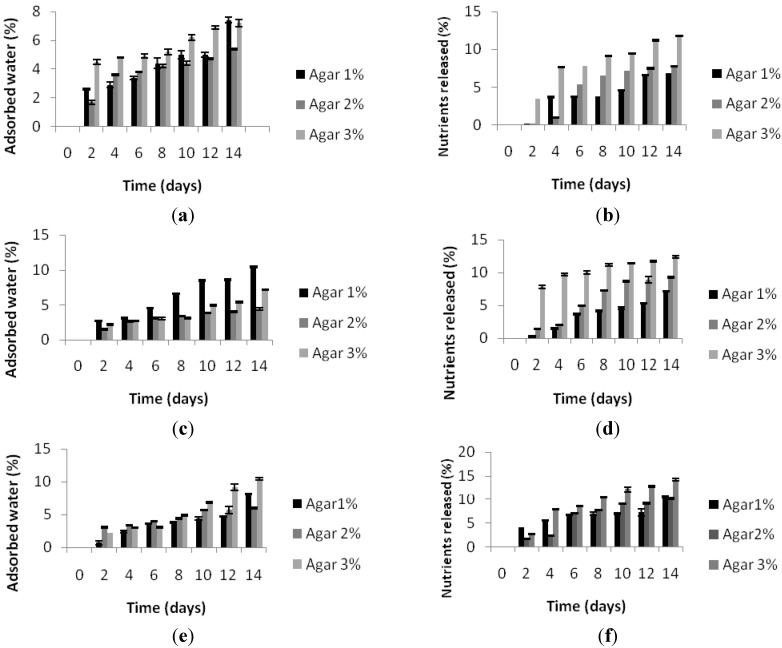

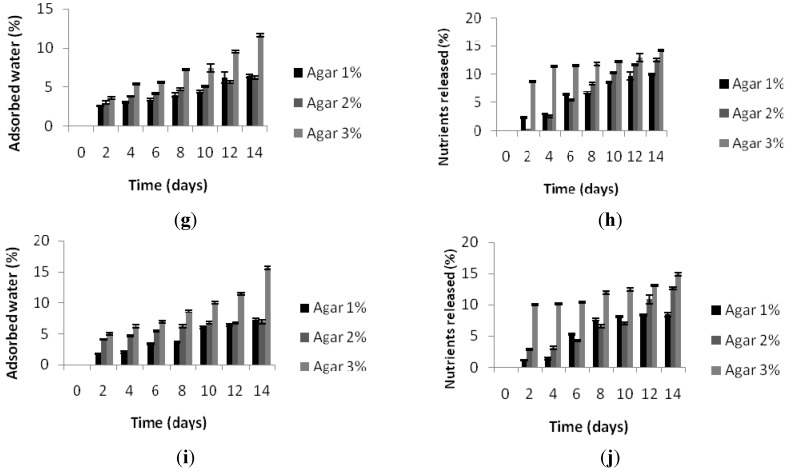
(**a**,**c**,**e**,**g**,**i**) percentage of water adsorbed by biocomposites made with agar 1%–2%–3% and containing nutrients 5%–10%–15%–20%–25%; (**b**,**d**,**f**,**h**,**j**) percentage of nutrients released by biocomposites with agar 1%–2%–3% and containing nutrients 5%–10%–15%–20%–25%.

In general, the percentage of water absorbed by biocomposites increased with the time of immersion and the percentage of nutrients employed. Figure 2a,c,e,g,i shows how agar 2%-based biocomposites show the best behavior in water, in terms of swelling, with respect to agar 1% and 3%. Indeed, biocomposites made of agar 2% absorbed less water with respect to biocomposites made of agar 1% and 3%, at any nutrient concentration. The amount of absorbed water increased according to the increase in the percentage of nutrients present in the biocomposites. In any case, water absorption was quite limited. In both 1% and 2% agar-based biocomposites, the percentage of absorbed water reached a maximum value of 10% after 14 days of immersion, while in the 3% agar-based biocomposites, it reached a value of 15% after 14 days of immersion. The agarose molecules that constitute the highly gelling fraction of agar have a high molecular weight due to the presence of left-handed triple helices that enable the polymer to generate a strong and stable gel, which can accommodate and firmly bind water molecules in thefree inter- and intra-molecular interstices [25], a phenomenon that could explain the greater degree of absorption of water in the biocomposites based on 3% agar.

#### 2.2.2. Nutrient Release

In spite of the importance of assessing nutrient release, there is still no standard method to determine it. In this study, we evaluated the dry matter loss after water immersion and indicate it as nutrient release. Nutrient release Figure 2b,d,f,h,j appeared to be proportional to the nutrient percentage in the biocomposites, with the exception of biocomposites based on agar1%,whichreleased less nutrients at 25% than nutrients at 15% and 20%. Moreover, nutrient release appeared to be proportional to the percentage of agar in the biocomposites. This outcome is in disagreement with observations made by Partridge and Southgate (1999) [26], who reported that nutrient release increased with decreasing binder concentration. However, it is possible that such a discrepancy may be attributed to the ample variability in feed ingredients and manufacturing technologies employed. It can be hypothesized that the greater degree of nutrient release from biocomposites based on agar 3% could be attributed to the ability held a higher amount of water that consequently determines thelower stability of the nutrients inside the three-dimensional networks of the gel. In 1% agar-based biocomposites, the lower amount of water held could conversely explain the higher stability of nutrients and, therefore, the lower nutrient release with respect to the 3% agar.Only in one case, that is biocomposites made of agar 1% with nutrients 10%, was the amount of water held higher than biocomposites made of agar 3%. Unfortunately, we do not have an explanation for such a discrepancy. Nevertheless, we cannot rule out the possibility that agar could undergo degradation during water incubation, thus altering the amount of nutrients released. 

#### 2.2.3. Granulometric Analysis

Granulometric analysis allows the monitoring of particles released by the biocomposites in water and the measurement of the diameter of the particles expressed as derived diameter. The low angle laser light scattering technique has been long employed in our laboratory to determine the water stability of feed for aquatic species [4,6,7,8,27]. The diameters of particles released by the biocomposites are continuously monitored over time, providing a time-course indication about the water stability as a function of the released particle diameter inasmuch as biocomposites that disaggregate into small particles are less stable in water than biocomposites that disaggregate into particles of a larger diameter. In Table 3 are reported the derived diameter of agar-based biocomposites at different percentages of nutrients.

The diameter of the released particles was quite constant throughout the experiment, ranging from about 10 to about 400 µm. All agar percentages behave well with all of the percentages of nutrients employed. The outcome is in agreement with previous observations carried out using the low angle laser light scattering technique to determine the diameter of particles released by agar-based biocomposites specifically designed for sea urchin feeding, which proved to have good water stability at least up to seven days [27].

**Table 3:** (**a**) Derived diameters (μm) of particles released in water at time intervals up to 14 days of biocomposites manufactured with percentages of nutrients of 5% and 10%; (**b**) derived diameters (μm) of particles released in water at time intervals up to 14 days of biocomposites manufactured with percentages of nutrients of 15% and 20%.

**(a)**						
**Days**	**Derived Diameters (μm)**			**Derived Diameters (μm)** **Nutrients 10%**		
	**Nutrients 5%**			**Nutrients 10%**		
	**Agar 1%**	**Agar 2%**	**Agar 3%**	**Agar 1%**	**Agar 2%**	**Agar 3%**
2	46.24 ± 0.02	13.82 ± 0.03	44.50 ± 0.22	54.41 ± 0.02	269.71 ± 0.41	108.35 ± 0.04
4	284.28 ± 0.11	57.44 ± 0.02	56.91 ± 0.23	304.51 ± 0.32	223.34 ± 0.32	171.62 ± 0.12
6	272.58 ± 0.04	160.62 ± 0.14	121.64 ± 0.02	251.39 ± 0.44	326.05 ± 0.19	206.51 ± 0.22
8	260.88 ± 0.04	162.90 ± 0.22	141.17 ± 0.02	204.66 ± 0.34	356.84 ± 0.24	142.19 ± 0.32
10	245.33 ± 0.12	176.49 ± 0.03	181.60 ± 0.02	287.82 ± 0.27	242.10 ± 0.12	64.83 ± 0.28
12	239.62 ± 0.02	286.53 ± 0.05	310.29 ± 0.02	324.26 ± 0.78	248.48 ± 0.52	67.62 ± 0.34
14	233.92 ± 0.12	255.93 ± 0.11	333.36 ± 0.02	339.68 ± 0.05	140.13 ± 0.62	70.42 ± 0.25
**(b)**						
**Days**	**Derived diameters (μm)**			**Derived diameters (μm)**		
	**Nutrients 15%**			**Nutrients 20%**		
	**Agar 1%**	**Agar 2%**	**Agar 3%**	**Agar 1%**	**Agar 2%**	**Agar 3%**
2	317.48 ± 0.32	121.37 ± 0.04	109.40 ± 0.08	277.23 ± 0.04	258.04 ± 0.14	245.63 ± 0.18
4	335.00 ± 0.22	224.71 ± 0.24	327.59 ± 0.18	323.17 ± 0.11	218.70 ± 0.29	211.55 ± 0.21
6	303.12 ± 0.12	242.44 ± 0.14	218.19 ± 0.13	349.98 ± 0.12	295.13 ± 0.30	227.21 ± 0.24
8	217.69 ± 0.02	271.51 ± 0.12	114.81 ± 0.10	375.91 ± 0.12	265.84 ± 0.34	258.74 ± 0.34
10	306.61 ± 0.23	119.16 ± 0.05	153.46 ± 0.12	364.81 ± 0.09	236.55 ± 0.27	290.28 ± 0.27
12	280.82 ± 0.11	186.35 ± 0.11	192.12 ± 0.09	273.88 ± 0.09	330.72 ± 0.24	286.87 ± 0.38
14	238.41 ± 0.11	254.70 ± 0.12	356.19 ± 0.08	231.23 ± 0.22	188.70 ± 0.38	106.41 ± 0.44

#### 2.2.4. Quercetin Release

**Figure 3:**
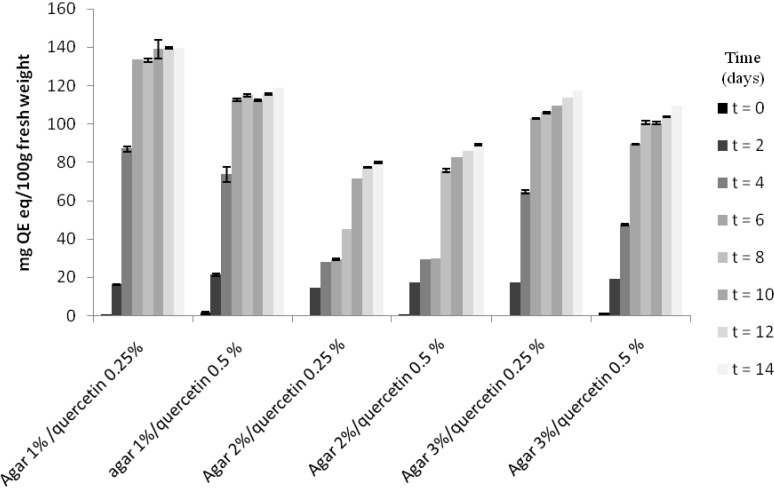
Quantity of quercetin (mg QE eq/100 g fresh weight) released by biocomposites made with agar 1%–2%–3% and containing nutrients 5%. Fresh weight refers to biocomposites formulated without any treatment before analysis.

Release of nutrients is an important feature of feed, since they can function as attractant molecules. Indeed, a certain degree ofleaching should always be allowed. In this study, we used quercetin to monitor the amount of small molecule leaching and chose quercetin due to its characteristics. Akroum *et al.* (2010) [28] tested the antibacterial activity of 11 flavonoids extracted from some medicinal plants and concluded that quercetin was the most interesting compound for all of the tested activities. Thus, quercetin could be usefully employed to improve both biocomposites’ shelf-life and the health performances of the farmed species due to its antioxidant and anti-microbial activities. The concentration of quercetin released by biocomposites in water is reported in Figure 3.

The trend of quercetin release was similar in all agar-based biocomposites. Quercetin release in water increased sharply during the first four days in 1% and 3% agar-based biocomposites, reaching values of respectively 87.01 ± 0.33 mg/100 g fresh tissue (quercetin 0.25%) and 73.75 ± 0.85 mg/100 g fresh tissue (quercetin 0.50%) in 1% agar-based biocomposites and 64.58 ± 0.02 mg/100 g fresh tissue (quercetin 0.25%) and 47.45 ± 0.03 mg/100 g fresh tissue(quercetin 0.50%) in 3% agar-based biocomposites. Slightly different was the behavior of 2% agar-based biocomposites that released a lower concentration of quercetin (27.80 ± 0.02 and 29.28 ± 0.02 mg/100 g fresh tissue for respectively quercetin 0.25% and 0.50%) up to the sixth day. From the eight day on, the quercetin concentrations released in water increased, but never as much as the values showed by 1% and 3% agar-based biocomposites. Again 2% agar-based biocomposites showed the best performances in terms of water behavior. As expected, the release of quercetin follows the same trends of nutrients.

## 3. Materials and Methods

### 3.1. Biocomposites Formulation and Preparation

The following biopolymers were employed: agar, sodium alginate and κ-carrageenan, supplied by Sigma (St. Louis, MO, USA). Biocomposites were first produced with varying percentages of nutrients (5%–25%) and polymers (1%–2%–3%), adding CaCl_2_ either alone or as a solution (0.5%–1%) to sodium alginate and κ-carrageenan as a networking agent.The best results were obtained with 0.5%of powdered CaCl_2_; therefore, the following descriptions refer to such parameters. All possible biopolymer combinations were tested in order to select those with the best network strength. The feed used for the nutrients was formulated on the basis of the current literature on aquatic species (Table 4).

Quercetin (Sigma-Aldrich Co., St. Louis, MO, USA) (0.25%–0.5%) was added to all biocomposites for its antimycotic and antioxidant properties. Biocomposites were manufactured by the following procedure: each biopolymer in powder form was dissolved to the desired percentage in tap water and heated to the boiling point on a magnetic plate, stirring constantly. The solution was allowed to boil for a few minutes and was then cooled at RT to a temperature of 45 ± 5 °C. Nutrients in powder form and CaCl_2_ (only in the presence of alginate and carrageenan) were then added to the mixture while stirring vigorously. The biocomposite was then poured into Petri capsules and allowed to cool at RT for 12 h. The round biocomposites (1-cm high, 4-cm diameter) were then removed from the Petri capsules and used for the further analyses. Network strength was subjectively evaluated in accordance with the terminology used by Pearce *et al.*, (2002) [22]: 1, very good consistency; 2, good consistency; 3, weak consistency; 4, inconsistent. Samples with “weak consistency” and “inconsistent” were discarded and were not considered for further trials. A complete list of manufactured biocomposites is provided in Table 4.

**Table 4:** Feed ingredients used in biocomposites.

Ingredient	Dry Weight (%)
Fish meal	19.00
Legume meal	27.00
Corn meal ^a^	24.00–25.00
Algae meal	25.00
Fish oil	1.80
Mineral mix	0.10
Vitamin mix	0.10

^a^ Percentage adjusted depending on the percentage of binder employed.

### 3.2. Behavior in Water of Biocomposites

#### 3.2.1. Swelling

Samples of round biocomposites listed in Table 4 among those selected for further trials were soakedat 20°C in water for up to 14 days. A round biocomposite was placed in a beaker and submerged with 200 mL of tap water. Only biocomposites with a network strength of 2 or less according to the terminology used by Pearce *et al.* (2002) [22], were tested for swelling. The swelling degree was expressed as a percentage of the increase in weight against the initial weight of the biocomposites and was calculated using the following equation:

water uptake (%) = [(W_f_ − W_0_)/W_0_] × 100

where W_0_ and W_f_ are the initial and final weight, respectively. Samples were recovered every two days and re-weighed in order to assess weight gain. All testswerecarried out in triplicate.

#### 3.2.2. Nutrients Release

To evaluate nutrients release, quantitative analysis was performed at 20 °C by evaporating the water in which the biocomposites were immersed. At time intervals of 2 days, up to 14 days, the amount of nutrients deposited on the bottom was determined by ponderal analysis according to A.O.A.C 2007 (Association of Official Agricultural Chemists) [29]. For each analysis, three samples for each type of biocomposites were employed.

#### 3.2.3. Granulometric Analysis

The diameter of nutrients released in water was measured by immersing samples of each biocompositi up to 14 days. For each analysis, three samples for each type of biocomposites were employed. After the required immersion time, samples were recovered, freeze-dried for 24 h and re-weighed. The recovered liquid was analyzed by monitoring the released particles in water during immersion. Particle size distributions were measured by LALLS (low angle laser light scattering technique) using Master Sizer Model S equipped with Malvern Application Software Version 1.1.a (Malvern Instruments, Malvern, UK) and fitted with a Small Volume Presentation Unit, as described in Volpe *et al.* (2008) [6]. The refractive index of samples and of dispersants was measured using an Optech Model RM Abbe refractometer at a temperature of 20 °C and white light.Specifically, LALLS and laser diffraction are based on the interaction of light-particle, called diffraction, which, as a physical principle, produces a dispersion of light on the ends of the particles with an angle inversely proportional to the size of the particle invested. After having focused on the center of a light-multi element detector, the effect of dispersion leading to signals on the elements is not central to the detector. Among the technical characteristics of the laser granulometer, there is an optics-unique and fixed measuring range: 0.02 to 2000 µM.

#### 3.2.4. Quercetin Release 

To evaluate quercetin leaching in water, biocomposites were submerged in water at 20 °C. For each analysis, three samples for each type of biocomposite were employed. At different time intervals (2 days up to 14 days), an aliquot of water (1 mL) was taken and employed to determine the quercetin concentration by spectrophotometric analysis. After the quercetin measurement, the water aliquot was returned to the beaker to keep thewater volume constant throughout the experiment. Quercetin absorbance was measured at 515 nm using a Beckman Coulter model. DU 730 Spectrophotometer (Indianapolis, IN, USA). The results were expressed as mg of quercetin equivalents/100 g fresh weight.

### 3.3. Statistical Analysis

Data were analyzed by one-way ANOVA, and the significant difference was determined at the 0.05 level by Duncan’s multiple range test. All analyses were performed with the StatSoft, Inc., STATISTICA data analysis software system, Version 8.0.

## 4. Conclusions

The feeding of aquatic animals stimulates the interest of researchers towards improving feed formulation. The feed must first be able to meet the different nutritional requirements in such a way as to ensure a degree of optimal growth; secondly, the critical issues related to the management of aquaculture systems must be considered, so as to minimize waste and pollution. In this experimental work, attention was focused on a particular aspect of the preparation of feed with structural characteristics, so as to prevent or at least minimize the release of nutrients in the water. To achieve these objectives, biopolymer-based feeds have been formulated, using macromolecules found in large quantities from natural renewable sources that, thanks to the peculiarity of forming hydrogels, are able to incorporate the nutrients in the three-dimensional network conferring firmness and stability to the feed. This study showed that the use of the agar-based biocomposites has a great degree of stability in water; this is in full agreement with the gelling characteristics of the polymer, which also in low percentages allows one to obtain three-dimensional networks that are strongly interpenetrated and resistant, so as to minimize the absorption of water and the release of nutrients.

Our results confirm the suitability of agar as a binder for biocomposites and provide a positive contribution to aquaculture.
